# Effect of Emotional States on EEG-Based Biometric Identification: A Comparative Study of Classifiers

**DOI:** 10.3390/bioengineering13060689

**Published:** 2026-06-16

**Authors:** Carolina Duque-Mejia, Camilo Zapata-Hernandez, Eduardo Duque-Grisales, Leonardo Serna-Guarin, Gustavo Lodoño-Ossa, Miguel A. Becerra

**Affiliations:** 1 Faculty of Engineering, Institución Universitaria Pascual Bravo, Cra. 73 #73A, Medellín 520001, Colombia; caroduquegm@gmail.com (C.D.-M.); juan.zapatah@pascualbravo.edu.co (C.Z.-H.); 2 Faculty of Business Studies, Institución Universitaria Esumer, Carrera 28 No. 19-24, Medellín 520001, Colombia; gustavo.londono@esumer.edu.co; 3 Faculty of Engineering, Instituto Tecnológico Metropolitano, Calle 73 No. 76A-354, Medellín 520001, Colombia; leonardoserna@itm.edu.co

**Keywords:** biometric identification, classifier fusion, EEG signal, machine learning, signal processing

## Abstract

Electroencephalographic (EEG) signals have been extensively studied for emotion detection and, more recently, as an alternative for biometric identification and authentication. Biometric methods based on physiological signals are a non-conventional approach for personal identification, and their study is currently considered an open research field. However, EEG-based biometric systems face several challenges, including the influence of emotional states, which can affect their performance. This study evaluates the effect of emotional states on the performance of an EEG-based biometric system. Four widely used databases for biometrics and emotion recognition (DEAP, MAHNOB, SEED, and LUMED-2) were selected for analysis. Feature extraction was performed using multiple strategies in the time, frequency, and time–frequency domains. The performance of various classifiers—support vector machine (SVM), random forest (RF), artificial neural networks (ANN), and k-nearest neighbors (K-NN)—was evaluated separately. Furthermore, stacking was used as a classifier fusion method. Explicit modeling of emotional states contributed to improving classifier performance. The best model based on classifier fusion achieved an accuracy of 95.73 ± 1.83%. These results indicate that incorporating information about emotional state into EEG-based biometric systems can contribute to the development of more robust and realistic identification solutions.

## 1. Introduction

Historically, methods for human identification have relied on passwords, access cards, and personal identification numbers (PINs). While these methods are commonly employed, they are accompanied by notable vulnerabilities, including the potential for theft, loss, or human errors [1]. Biometric identification has emerged as a promising alternative to reducing the limitations of traditional methods [2,3]. This approach leverages the unique physical characteristics and physiological signals of individuals, such as fingerprints, iris patterns, facial features, voice, and physiological signals like electroencephalograms (EEG), electromyograms (EMG), and electrocardiograms (ECG), among others [4,5]. EEG is a non-invasive technique that records the electrical activity of the brain using electrodes placed on the scalp [6,7]. The electrical activity generated by ionic current flows within neurons creates a rich and complex data source that can be utilized for applications like biometric identification. This is due to the inherent difficulty in duplicating or manipulating these signals [8,9]. Although EEG signals provide greater security than traditional methods, they also present several challenges, including artifacts and noise that affect signal quality, as well as variability associated with fatigue, cognitive load, and emotional states [10,11].

EEG-based emotion recognition studies have shown that emotional states can be represented through discriminative EEG patterns [12]. This evidence supports the need to explicitly consider emotional variability in EEG-based biometric identification systems, since such variability may affect their stability, reliability, and performance [13].

In addition, EEG-based emotion recognition studies have shown that emotional states can be represented through discriminative EEG patterns, supporting the need to explicitly consider emotional variability on biometric identification systems.

In [14] used the Biometric EEG Dataset (21 individuals) and deep learning models (ResNet, Inception, and EEGNe) as classifiers. EEG signals were recorded using an Emotiv EPOC+ device. The achieved accuracies were 63.21% for ResNet, 70.18% for Inception, and 86.74% for EEGNet. In [15] developed a system using a proprietary database (6 men and 6 women aged between 24 and 45). Classifiers such as Support Vector Machines (SVM), K-Nearest Neighbors (K-NN), and Linear Discriminant Analysis (LDA) were implemented, yielding an accuracy of nearly 100%. To enhance system usability, the effect of reducing the number of electrodes on classifier performance was assessed. The system employed electrodes AF3, AF4, F7, and F8 alongside a K-NN classifier, demonstrating an accuracy of 99.0 ± 0.8%. Barayeu et al. in [16] investigated the effects of a dimensionality reduction method on a biometric system based on EEG signals. This study evaluated an inception-like network, a VGG-like deep learning neural network (NN), and principal component analysis (PCA), using SVM as the classifier. The highest accuracy of 95.64% was achieved by utilizing a 64-channel PCA as a dimensionality reduction strategy. In [17] evaluated several classifiers, including SVM, K-NN, and Random Forest (RF). The results evidenced that the SVM and AdaBoost classifiers achieved accuracies of 85.94% and 99.83%, respectively. In a similar study [18], Tatar employed classifiers including SVM, K-NN, ANN, RF, and Gradient Boosting, with Random Forest achieving the highest accuracy of 98.14%. The authors suggested that future research should investigate how environmental conditions, and emotional states affect model performance. Additionally, another analysis found SVM to be the top performer, though it did not consider the influence of emotional states. A variety of databases, dimensionality reduction techniques, feature extraction methods, and classifiers have been assessed in EEG-based recognition tasks, yielding promising results [19]. However, research on the impact of emotions on EEG-based biometric identification systems remains limited. Vahid & Arbabi [20] investigated which features produced stable outcomes irrespective of emotional states. They extracted 54 features from 32 EEG channels across different emotional conditions, discovering that features from five channels consistently delivered reliable results. The variance of wavelet coefficients within the frequency range of 32–64 Hz, the amplitude of the spectrum at central frequencies within the 25–45 Hz range, and the total absolute area emerged as the most effective features. The identification rates using the SVM classifier and the selected features ranged from 88% to 99%. In a related study, Rakshe et al. [21] developed a biometric system based on EEG under various emotional states, using the DEAP database. They extracted 76 features for each channel, which included statistical characteristics from the temporal domain, frequency domain, entropy, fractal and chaos theory features, spectral analysis, and shape features. The study evaluated several classifiers: bagging classifier (BC), RF, gradient boosting machine (GBM), XGBoost, light gradient boosting machine (LightGBM), and categorical boosting (CatBoost). The accuracy rates for BC, RF, GBM, XGBoost, LightGBM, and CatBoost were 84%, 89%, 76%, 81%, 85%, and 91%, respectively. Emotional states are crucial in our everyday lives and are shaped by our emotional experiences over time. Consequently, everyone’s reactions to specific stimuli can differ, which is valuable for biometric identification using EEG signals [22]. To the authors’ knowledge, the influence of emotional states, datasets, and classifiers has not been extensively examined. On the other hand, recent studies have also explored more advanced EEG modeling approaches, including normalized mutual information-based feature representations, graph attention convolutional neural networks, and deep learning architectures, for EEG-based emotion recognition and related neurophysiological classification tasks [23,24,25].

The motivation of this work is that EEG-based biometric systems are expected to operate reliably under real-world variability, yet emotional states can modulate EEG patterns and potentially degrade identification performance. Although EEG biometrics has been widely studied, the effect of emotion-induced variability on identification accuracy and robustness has not been systematically assessed across commonly used classifiers and across multiple EEG databases. Consequently, this study is driven by the need to quantify how changes in emotional state influence EEG-based biometric identification, providing evidence that can guide the design of more robust systems. Therefore, this study aims to assess how these factors—dataset and classifier—affect the performance of biometric systems.

The main contributions of this study are as follows: (i) the effect of emotional states on EEG-based biometric identification is evaluated across four commonly used EEG databases, namely DEAP, MAHNOB, SEED, and LUMED-2; (ii) a comparative analysis of several machine learning classifiers, including RF, ANN, LR, SVM, and K-NN, is performed under emotional and non-emotional feature settings; (iii) the relevance of time-domain, frequency-domain, time–frequency, entropy-based, and spatial features is analyzed using ReliefF-based feature selection; and (iv) a classifier fusion strategy based on stacking is evaluated to determine whether combining classifiers improves biometric identification performance under emotion-induced EEG variability.

We evaluated a set of commonly used machine learning algorithms for EEG classification, including RF, ANN, LR, SVM, and K-NN, to provide a comprehensive comparative analysis. This choice was motivated by the fact that, in most prior studies, the impact of emotional states on the performance of these classifiers has not been systematically assessed. In addition, using these algorithms allowed us to examine their robustness across multiple EEG databases.

The manuscript follows a standard scientific structure. Section 2 describes the EEG databases, signal preprocessing, feature extraction, classification algorithms, and evaluation metrics. Section 3 presents and discusses the experimental results, emphasizing the comparative performance of the evaluated classifiers across databases and emotional states. Finally, Section 4 presents the conclusions and future research directions.

## 2. Materials and Methods

**Figure 1:**
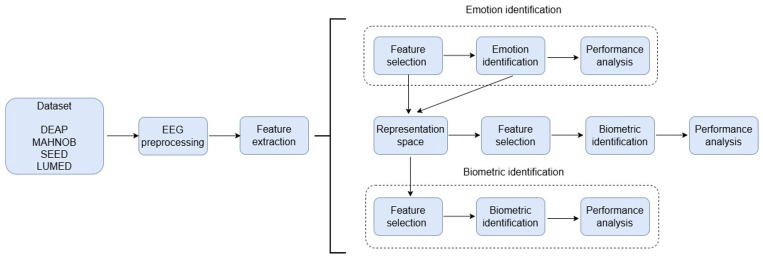
Proposed procedure.

Figure 1 shows the approach adopted for biometric identification based on EEG signals, considering multiple emotional states. Four databases were selected, which are commonly used in biometric identification and emotion recognition. Each database was analyzed independently to assess the variability and consistency of EEG signals across different subjects and emotions.

### 2.1. Database Description

#### 2.1.1. DEAP

This database contains EEG signals and peripheral physiological signals from 32 participants. Each participant viewed one-minute excerpts of music videos, which were rated based on arousal, valence, liking, dominance, and familiarity levels. The group comprised 50% females and 50% males. Their ages ranged from 19 to 37, with an average age of 26.9. In addition, the dataset includes the peripheral recordings, such as electrooculogram (EOG), four electromyography (EMG) signals from the zygomaticus major and trapezius muscles, galvanic skin response (GSR), blood volume pressure (BVP), temperature, respiration, and the frontal facial recordings from 22 participants. The signals were recorded on 32 channels at 512 Hz [26].

#### 2.1.2. MANHOB

The database involved a total of 27 participants, 11 men and 16 women. EEG signal recordings were registered across 32 channels at 256 Hz. In addition, face and body recording videos were made using 6 cameras (60 f/s). The eye gaze was collected at 60 Hz; audio recordings were made at a sampling rate of 44.1 kHz. A total of 20 videos were recorded from which emotional keywords were obtained. These included measures of arousal, valence, dominance, and predictability, using a range of evaluations from 1 to 9 [27].

#### 2.1.3. SEED

This database consists of EEG signals from 7 men and 8 women aged between 23 and 27 years. To evoke positive, negative, and neutral emotions, 15 clips from Chinese films were selected as stimuli. Each experiment consists of 15 trials, and the EEG signals were recorded using a cap that complies with the international 10–20 system for 62 channels. The signals were preprocessed and filtered at 200 Hz. Finally, each signal is labeled as follows: −1 for negative, 0 for neutral, and +1 for positive emotions [28].

#### 2.1.4. LUMED-2

Loughborough University Multimodal Emotion Database-2 (LUMED-2) is a novel multimodal dataset that contains simultaneous data collected from 13 participants (6 females and 7 males). The participants were exposed to visual stimuli designed to elicit specific emotions. The total duration of all stimuli is 8 min and 50 s, consisting of short video clips selected from the internet. After each session, participants were asked to label the clips based on the emotional state they experienced while viewing them. Three different emotions were identified from the labeling: “sad”, “neutral”, and “happy”. The facial expressions of participants were recorded using a webcam with a resolution of 640 × 480 and a frame rate of 30 frames per second. Additionally, the EEG signals for each participant were recorded using an 8-channel ENOBIO wireless EEG device (Neuroelectrics, Barcelona, Spain), which has a temporal resolution of 500 Hz. The EEG signals were filtered for the frequency range of 0 to 75 Hz, and baseline subtraction was applied for each window. Finally, the GSR of participants was collected using a Bluetooth-powered EMPATICA E4 wristband (Empatica Inc., Cambridge, MA, USA) [29].

### 2.2. Preprocessing

All signals from DEAP, SEED, LUMED, and Mahnob databases were normalized to a range of 0 to 1. Additionally, the signals were bandpass-filtered using specific cutoff frequencies for each frequency band: delta (0.5–4 Hz), theta (4–8 Hz), alpha (8–13 Hz), beta (13–30 Hz), and gamma (30–45 Hz). The signals were divided into several non-overlapping windows. Each 1 min recording was segmented into 256 windows, with a step of 128 samples between consecutive windows. Therefore, consecutive windows shared 50% of their samples. The number of windows generated for each dataset depended on the sampling frequency, recording duration, number of trials, and number of subjects available in each database.

### 2.3. Feature Extraction

In the time domain, several statistical measures were calculated, such as mean, variance, standard deviation, correlation coefficient. In addition, entropy-based features (Shannon entropy and sample entropy) were included because they provide quantitative descriptors of signal uncertainty and complexity that have proven informative in EEG analysis and biometric applications. In the frequency domain, statistical measures were calculated based on the fast Fourier transform (FFT) results. Moreover, the discrete wavelet transform was employed to decompose the signal into five levels. This methodology facilitated the simultaneous analysis of extended intervals for low-frequency information and shorter intervals for high-frequency data. Several mother wavelets (Daubechies, Mexican Hat, and Symlet) were used for their inherent differences in capturing signal details at different frequency scales, which is crucial for distinguishing between emotions and biometric identification. Finally, the common spatial patterns (CSP) were used to obtain characteristics in the spatial domain.

### 2.4. Feature Selection

Feature selection in EEG signals was carried out using the ReliefF algorithm [30]. This method assesses the relevance of each feature by assigning weights according to its ability to distinguish between neighboring instances from the same and different classes. Specific selection criteria were established based on the importance scores assigned by the ReliefF algorithm. ReliefF is particularly useful when samples are similar but belong to different classes, since it evaluates feature relevance using nearest-neighbor comparisons.

In this study, ReliefF was selected as the feature selection method to maintain the compactness of the methodological process and focus on evaluating the impact of emotional states on EEG-based biometric performance. Systematic comparison with other feature selection techniques (e.g., filter-based, envelope-based, and integrated approaches) is left for future work.

We performed feature selection in two stages to support two complementary analysis settings. First, feature selection was carried out for the emotion-oriented pipeline to identify the most informative descriptors under the emotional-state setting. Second, feature selection was repeated for the integrated biometric pipeline, where emotion-related measures were combined with purely biometric features (i.e., biometric identification without explicit emotion descriptors) in order to re-estimate feature relevance on the merged feature space. This second round was necessary because the importance/ranking of features may change once emotion-related descriptors are introduced alongside biometric features, and our objective was to explicitly assess the contribution of emotion-related measures within the final biometric identification setting.

### 2.5. Classification Methods

To investigate how emotional states affect EEG-based biometric classification, we selected a set of widely used machine learning algorithms—Random Forest (RF), Artificial Neural Networks (ANN), Logistic Regression (LR), Support Vector Machines (SVM), and k-Nearest Neighbors (KNN)—to enable a comprehensive and fair comparative analysis. These models were intentionally chosen because they represent complementary decision principles (i.e., different inductive biases): SVM is a margin-based classifier that seeks a separating hyperplane in an *n*-dimensional feature space; KNN is a non-parametric, proximity-based method that assigns labels according to the nearest samples in the feature space; RF is an ensemble of decision trees that aggregates multiple weak learners to improve generalization; LR provides a simple probabilistic baseline for binary decisions; and ANN offers a flexible nonlinear function approximator. This diversity allows us to assess whether emotion-induced variability impacts performance consistently across classifier families, and to examine robustness across multiple EEG databases while keeping the evaluation aligned with common practice in EEG biometrics. In addition, we deliberately did not include more complex models that are often reported to achieve strong performance in EEG classification (e.g., deep learning architectures or gradient-boosted ensembles such as XGBoost). Our goal was not to maximize accuracy through model capacity, but to isolate and quantify the influence of emotional states on biometric identification performance under a comparable and widely adopted set of classifiers. For each dataset, the feature matrix was divided into training and testing subsets using a stratified partition strategy to preserve the proportion of classes in both sets. Specifically, 80% of the samples were used for training and 20% for testing. The same partitioning scheme was applied to all evaluated classifiers in order to ensure a fair comparison among models. Stratification was used to reduce class imbalance effects and to maintain comparable subject/emotion distributions across the training and testing subsets.

The main hyperparameters used for each classifier are summarized in Table 1. These parameters were selected to provide a stable and comparable baseline across datasets while avoiding excessive model complexity.

#### 2.5.1. K-Nearest Neighbors (K-NN)

The K-NN classifier is a simple and widely used distance-based method for classification tasks [17,31]. The KNN is a non-parametric technique that classifies new data points by considering the majority class among their ‘k’ nearest neighbors in the feature space. The underlying principle of KNN is that similar data points tend to be located close to one another in the space defined by their features [32].

#### 2.5.2. Artificial Neural Network (ANN)

ANNs are nonlinear classifiers designed to mimic the structures of the human neural system [33]. The most common structure of an ANN is the multilayer perceptron (MLP), which typically consists of an input layer, one or more hidden layers where neurons perform weighted summations along with activation functions, and an output layer that generates predictive outcomes. The weighted sum for a neuron is calculated as follows [34]:

(1) $$z_{j}=\sum_{i=1}^{n}w_{ji}x_{i}+b_{j}$$

where $w_{ji}$ are weights, $x_{i}$ are inputs (the i-th component of the input feature vector x), and $b_{j}$ is the bias.

#### 2.5.3. Support Vector Machines (SVM)

SVM is a margin-based classifier that separates samples by constructing an optimal hyperplane with the maximum margin between classes [35]. It achieves nonlinear classification using different kernel functions. It is widely used in EEG-based authentication [36]. Mathematically, assume the input dataset:

(2) $$x^{i}=\left(x_{1}^{i},x_{2}^{i},\ldots ,x_{n}^{i}\right)$$

This represents a realization of the random vector x. The function ϕ(·) maps the input vector into a transformed feature space, where a separating hyperplane can be constructed.

The SVM finds an optimal separating hyperplane defined by the weight vector w and the bias term *b*. The vector w determines the orientation of the hyperplane, whereas *b* controls its displacement with respect to the origin. These parameters are estimated during the training process by solving the SVM optimization problem, which maximizes the separation margin between classes. The following quantity is maximized (in this formulation, maximizing the margin is equivalent to minimizing $\frac{1}{2}{∥w∥}^{2}$ subject to the class-separation constraints):

(3) $$\rho =\min_{i}y^{i}\left(\langle w,\phi (x^{i})\rangle +b\right)$$

In this expression, ρ denotes the functional margin, and $y_{i}$ is a scalar class label associated with the training sample $x_{i}$, where typically $y_{i}\in \left\{-1,+1\right\}$. The operator 〈·,·〉 denotes the inner product between two vectors, and $\phi (x_{i})$ represents the mapping of the input vector into a transformed feature space. First, the inner product $\langle w,\phi \left(x_{i}\right)\rangle$ is computed; then the bias term *b* is added; finally, the result is multiplied by the scalar label $y_{i}$ to impose the class-separation constraint.

The margin represents the separation between the decision boundary and the closest training samples. In the SVM formulation, $\phi (x^{i})$ denotes the mapping of the input vector $x^{i}$ into a transformed feature space. The decision function before applying the sign operator is expressed as $\langle w,\phi \left(x^{i}\right)\rangle +b$, where w is the weight vector and *b* is the bias term. The signed distance from the transformed point $\phi (x^{i})$ to the decision boundary is given by the normalized decision function:

(4) $$d\left(x^{i}\right)=\frac{\langle w,\phi \left(x^{i}\right)\rangle +b}{∥w∥}$$

where ∥·∥ denotes the Euclidean norm. Therefore, ${∥x-z∥}^{2}$ represents the squared Euclidean distance between the vectors *x* and *z*.

In this study, a radial basis function kernel was used. This is defined as:

(5) $$K\left(x,z\right)=\exp\left({-\gamma ∥x-z∥}^{2}\right)$$

In the RBF kernel, γ is a kernel scale parameter that controls the influence of each training sample in the transformed feature space. A larger value of γ produces a more localized decision boundary, whereas a smaller value leads to a smoother boundary. Therefore, in this context, γ should not be confused with the SVM margin previously discussed.

Given a new point *x*, the label is assigned according to its relation to the decision boundary. The corresponding decision function is expressed as:

(6) $$f\left(x\right)=\mathrm{sign}\left(\langle w,\phi (x)\rangle +b\right)$$

#### 2.5.4. Random Forest (RF)

RF is a combination of predictive trees such that each tree depends on the value of a random vector sampled independently and with the same distribution for all forest trees [37]. For classification tasks, the final prediction of a random forest is determined by a voting process, where each tree in the forest outputs a class prediction, and the class with the majority of votes is considered the forest’s prediction [38].

#### 2.5.5. Data Fusion Approach

Stacking is a learning approach that combines the predictions made by several classifiers generated by different learning algorithms $L_{1},L_{2},\ldots ,L_{n}$. These classifiers are trained on the same training data $D_{\mathrm{train}}$, which contains examples of the form $S_{i}=\langle x_{i},y_{i}\rangle$, where $x_{i}$ is the feature vector and $y_{i}$ is the associated label [39].

#### 2.5.6. Performance Metrics

Finally, the metric used to evaluate the classifier was accuracy. This metric provides an overall measure of performance for classifiers. It is calculated as the ratio of correct predictions to the total number of predictions [40,41].

(7) $$\mathrm{Accuracy}=\frac{TP+TN}{TP+TN+FP+FN}$$

where TP, TN, FP, and FN denote true positives, true negatives, false positives, and false negatives, respectively.

### 2.6. Statistical Significance Test

In this study, statistical tests were used to determine significant differences in the ranking of the prediction model’s performance [42]. The Friedman test was selected as the nonparametric alternative to repeated-measures ANOVA. This test is suitable for comparing multiple models evaluated on the same datasets, particularly when the distribution of performance metrics is unknown or the assumption of normality cannot be guaranteed. The Wilcoxon signed-rank test was used to assess the dataset-level effect of including emotional states. For each EEG dataset, performance differences between models with and without emotional information were averaged across classifiers, avoiding the treatment of classifier–dataset combinations as fully independent observations. This nonparametric test was selected because it is suitable for paired comparisons without assuming normality. A significance level of α=0.05 was used [43].

## 3. Results and Discussion

### 3.1. Relief-Based Feature Selection Results

Table 2 shows the relevant features obtained with the *Relief* algorithm.

The best results were obtained from the wavelet transform features, highlighting Shannon entropy (ShEn), sample entropy (SampEn), Common Spatial Patterns (CSP), and the mean values of the EEG signals recorded from the Fp1, F7, and FC1 channels. These channels correspond to electrode positions in the international 10–20/10–10 EEG placement system; Fp1 and F7 are located in frontal scalp regions, whereas FC1 is located in a fronto-central scalp region.

The implementation of this feature selection method improves computational efficiency and contributes to a more accurate and robust analysis of EEG signals. The consistency in the relevance of these features across different datasets improves robustness and discriminant ability.

**Figure 2:**
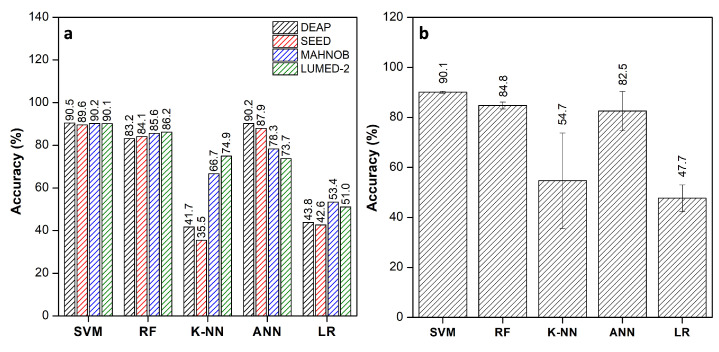
Performance of EEG-based biometric classifiers across multiple datasets. (**a**) Accuracy obtained by each classifier for the DEAP, SEED, MAHNOB, and LUMED-2 datasets. (**b**) Mean accuracy of each classifier across the evaluated datasets.

Figure 2a shows the performance of machine learning models on four datasets (DEAP, SEED, MAHNOB, and LUMED-2). The average accuracy for each model is shown in Figure 2b. SVM exhibits a higher average accuracy (90.1%), with a lower standard deviation (0.39%). In contrast, K-NN evidenced a higher standard deviation (19.08%) compared to the other models.

The Friedman test showed statistically significant differences among the evaluated classifiers, $\chi^{2}\left(4\right)=13.00$, p=0.0113. In addition, the ranking analysis indicated that SVM achieved the best average rank across the EEG datasets, meaning that it consistently obtained the highest relative performance, followed by RF and ANN.

The SVM demonstrated higher accuracy than other classifier models, as reported by Jalaly Bidgoly et al. [18]. This increased accuracy of the SVM can be attributed to the nonlinear nature of EEG signals. The radial basis kernel used in this study effectively maps these nonlinear features into a higher-dimensional space, facilitating better data separation and classification. In contrast, the KNN method evidenced lower accuracy compared to the other classifiers. This could be attributed to the presence of overlapping samples that typically occur near class boundaries [44].

### 3.2. Impact of Emotional States and EEG Classifiers’ Performance

**Figure 3:**
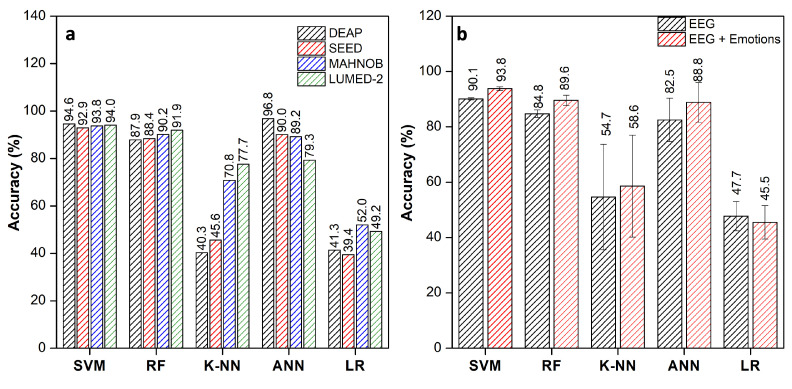
Performance of classifiers using EEG signals and emotional states. (**a**) Accuracy obtained by each classifier for the DEAP, SEED, MAHNOB, and LUMED-2 datasets when EEG features were combined with emotional-state information. (**b**) Comparison between the mean accuracy obtained using EEG features alone and EEG features combined with emotional-state information across the evaluated datasets.

Figure 3a shows the performance of machine learning models on four datasets, DEAP, SEED, MAHNOB, and LUMED-2, with an emphasis on emotions. The integration of emotional data improved the accuracy of all classification models. The SVM model evidenced an average accuracy of 93.82%, which is higher than other models (see Figure 3b). Incorporating emotions into biometric systems causes an increase in the average accuracy of most models compared to those that did not use emotional data. Finally, the linear regression evidenced a performance reduction compared to the model without the integration of emotions.

Emotional estates increased mean performance from 71.96% to 75.27% (+3.31 percentage points); however, the Wilcoxon signed-rank test was not significant, W=0.00, p=0.1250. This may be explained by the small number of independent datasets, which limits statistical power. Thus, the results should be interpreted as exploratory evidence.

The high accuracy and stability of SVM can be related to its ability to project input data into a higher-dimensional space, where a clear separation between classes can be achieved. In addition, the SVM is widely used to distinguish between emotional states due to its performance [45]. This suggests that SVM could be used to develop biometric systems based on EEG and emotional states. This study evaluated four databases with different characteristics, revealing different sampling strategies and protocols to induce emotions. In general, in this study, emotions caused an increase in classifier performance, which favors the development of robust systems [46]. The increase in the performance of classifiers could be related to emotional states, which are a complex experience, influenced by subjective cognition and the external environment. Given the same stimulus, each person could experience a different emotional state, which could be valuable in the field of biometric identification [47].

### 3.3. Effect of Classifier Fusion Method on System Performance

**Figure 4:**
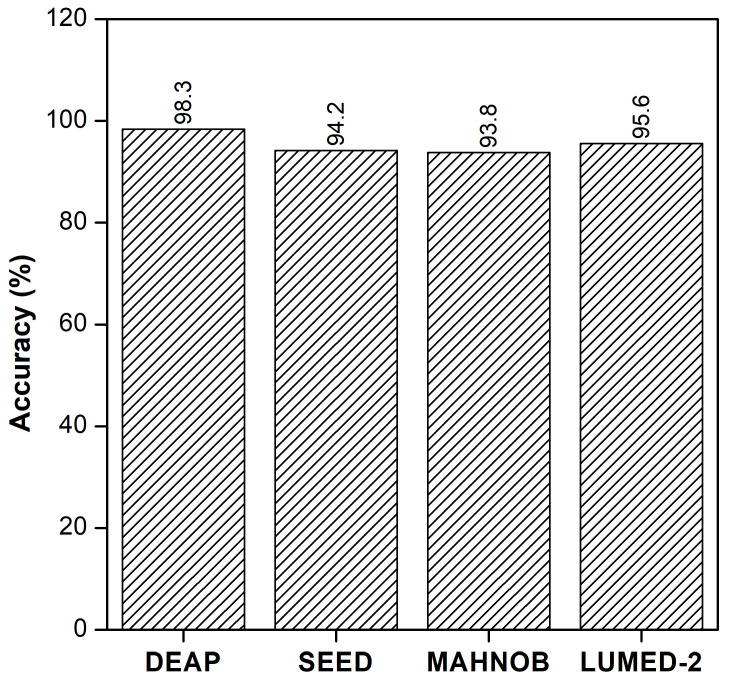
Performance of classifier fusion.

Figure 4 shows the performance of the classifier fusion. The highest accuracy (98.3%) was obtained with the DEAP dataset. The mean accuracy evidenced was 95.73 ± 1.83%, which outperforms individual classifiers.

This result suggests that, while the SVM individually showed better performance compared to the other classifiers, the stacking method favors an increase in the performance of the biometric system. Stacking allows errors in the models to compensate for each other because each classifier provides different information for the identification of people using EEG signals in various emotional states [48,49]. This strategy favors an increase in the performance of biometric systems.

Table 3 shows a comparative analysis of the results obtained in this study alongside those reported in the literature. Biometric systems that do not incorporate emotional factors for identification have demonstrated accuracy rates ranging from 82% to 100%, indicating that the proposed work is competitive. Recently, Ref. [21] used emotions to develop a biometric system based on EEG; however, they reported lower accuracy than what was achieved in this research.

To contextualize the comparison in Table 3, it is important to note that differences in the accuracy of identification between studies are strongly influenced by the characteristics of the database, the feature-extraction pipeline, and the evaluation protocol. For example, Rakshe et al. [21] extracted a large multi-domain feature set (e.g., statistical, time-domain, frequency-domain, entropy and fractal/chaos-related indices, spectral descriptors and shape measures) and evaluated several ensemble learners (e.g., RF and boost variants). Hernández-Álvarez et al. [50] explored both multi-class (e.g., SVM, RF, KNN) and one-class (e.g., Isolation Forest) authentication schemes, reporting best performance with anomaly-detection/ensemble approaches. Tatar [18] reported very high SVM performance using a different dataset and protocol, combining a set of statistical descriptors with Neighborhood Component Analysis for feature selection and also benchmarking a deep neural network; such design choices can substantially alter the effective feature space and, consequently, the behavior of SVM. Similarly, Kamaraju et al. [51] used signal decomposition (MVMD) and Fourier–Bessel spectral entropy features, together with kernel-based classification (e.g., cubic SVM). Therefore, the higher SVM accuracy reported by Tatar [18] should not be interpreted as a direct one-to-one improvement over our SVM results, since dataset properties (number of subjects, recording conditions, elicitation paradigm), preprocessing, feature definitions, and validation strategies differ and can produce large shifts in classifier performance.

On the other hand, Ref. [21] proposed a framework to evaluate an EEG-based biometric system under different emotional states using the DEAP database and four affective conditions (HAHV, HALV, LALV, and LAHV). They extracted a broad feature set per channel, including statistical, time-domain, frequency-domain, entropy-based, fractal, spectral, and waveform-shape descriptors, and reported accuracies of 84%, 89%, 76%, 81%, 85%, and 91% with bagging, RF, GBM, XGBoost, LightGBM, and CatBoost, respectively. The strong performance of CatBoost has been associated with effective regularization and robustness to feature interactions and multicollinearity, which can be beneficial when using large, partially redundant feature sets. Although both studies rely on DEAP, a direct comparison remains limited because differences in preprocessing, feature definitions, feature-selection strategy, validation protocol, and the use of boosting-based ensembles in [21] can substantially affect the resulting performance. Finally, Arnau-González et al. [53] used the SEED dataset to analyze how the feature extraction method influences classifier performance in EEG-based subject identification. Three feature representations were compared: Mel Frequency Cepstral Coefficients (MFCC), Auto-Regression Reflection Coefficients (ARRC), and Power Spectral Density (PSD). The best results were obtained with MFCC features, where SVM achieved an accuracy of 79% using a linear kernel. In contrast, PSD-based features showed lower performance, highlighting that the EEG feature representation has a significant effect on the discriminative capability of the classifier.

The performance of a biometric system depends on multiple factors such as the database, classifiers, and feature extraction process, among others. Particularly, in the recognition of individuals through EEG signals and mentions, the extraction of features is relevant to the identification process because of the correlations between the information of the extracted features and the emotional states [54,55]. It is possible that the features used in this study for training the models could explain the results.

## 4. Conclusions and Future Research Directions

In this study, emotional states favored an increase in the performance of individual classifiers. The SVM showed higher average accuracy (93.82%) and greater robustness compared to the other classifiers. SVM using the radial basis kernel maps the features to a higher-dimensional space, favoring the separation between classes. On the other hand, data fusion showed a higher accuracy (95.73%) compared to SVM. This study contributes to the understanding of the effect of emotional states on EEG-based biometric systems.

Although cross-subject validation is commonly used in EEG emotion recognition studies, the present work focuses on EEG-based biometric identification, where subject identity is the target class. Therefore, the experimental protocol is not directly equivalent to cross-subject emotion recognition settings, in which the objective is to classify emotional states from unseen subjects. Future work should explore complementary evaluation protocols, such as cross-session, cross-dataset, and open-set identification scenarios, to further assess the generalization capacity of EEG-based biometric systems under emotion-induced variability. Additionally, future work should incorporate an information quality assessment approach into the proposed EEG-based biometric identification framework [56]. This perspective would make it possible to evaluate not only the final classification performance, but also the quality of the input EEG signals, the reliability and traceability of the extracted features, the consistency of the data, the uncertainty introduced during preprocessing and feature fusion, and the influence of emotional states as contextual information. In this sense, the JDL data fusion model could be adopted as a reference framework to structure the biometric process into functional levels, including source preprocessing, feature and object refinement, contextual assessment of emotional states, process refinement, and user/system feedback. This quality-oriented perspective may contribute to the development of more robust, explainable, and adaptive EEG-based biometric systems, especially under variable emotional and acquisition conditions.

## Figures and Tables

**Table 1:** Main hyperparameters used for the evaluated classifiers.

Classifier	Hyperparameters
k-NN	k=5 nearest neighbors; Euclidean distance metric; uniform weighting; brute-force neighbor search.
SVM	Radial basis function (RBF) kernel; regularization parameter C=1.0; kernel coefficient γ=scale; tolerance $=10^{-3}$; maximum iterations not limited.
RF	Number of trees =100; Gini impurity criterion; bootstrap aggregation enabled; maximum tree depth not restricted; minimum samples per split =2; minimum samples per leaf =1; number of features considered at each split $=\sqrt{n_{\mathrm{features}}}$.
LR	L2 regularization; regularization strength C=1.0; solver = lbfgs; maximum iterations =1000; tolerance $=10^{-4}$.
ANN	Multilayer perceptron architecture; one hidden layer with 100 neurons; ReLU activation function; Adam optimizer; initial learning rate =0.001; batch size =32; maximum iterations =1000; L2 penalty α=0.0001.

**Table 2:** Relevant features selected using the ReliefF feature selection algorithm.

Dataset	Features			
DEAP	ShEn	SampEn	Mean	CSP
SEED	ShEn	CSP	EnSamp	Mean
MANHOB	ShEn	SampEn	CSP	Mean
LUMED-2	ShEn	SampEn	CSP	Mean

**Table 3:** Comparative analysis of the results obtained in this study alongside those reported in the literature.

Reference	Dataset	Classifier/Accuracy	Emotion Used?
[21]	DEAP	RF (89%)	Yes
		Gradient Boosting (89%)	Yes
		Extreme Gradient Boosting (76%)	Yes
		LightGBM (81%)	Yes
		CatBoost (85%)	Yes
		Bagging (91%)	Yes
[50]	Collected by the authors	RF (82.3%)	No
[18]	Physionet	Decision Tree (98.63%)	No
		SVM (99.91%)	No
		RF (100%)	No
[51]	Dataset name	KNN (93.4%)	No
[52]	DEAP	RF (76%)	Yes
		SVM (68%)	Yes
[17]	DEAP	SVM (99%)	No
		AdaBoost (99%)	No
[53]	SEED	SVM (79%)	Yes
This study	DEAP, MAHNOB, SEED, LUMED-2	SVM (93.82%)	Yes
This study	DEAP, MAHNOB, SEED, LUMED-2	Data fusion approach (95.73%)	Yes

## Data Availability

The original contributions presented in this study are included in the article. Further inquiries can be directed to the corresponding authors.
